# A TELEHEALTH DELIRIUM COACHING INTERVENTION FOR FAMILY CAREGIVERS OF OLDER ADULTS WITH ALZHEIMER’S DISEASE

**DOI:** 10.1093/geroni/igz038.3108

**Published:** 2019-11-08

**Authors:** Deborah A D’Avolio, Andra Opalinski, David Newman

**Affiliations:** Florida Atlantic University, Boca Raton, Florida, United States

## Abstract

The purpose of this study is to develop and evaluate the feasibility of a telehealth coaching intervention for delirium prevention among family caregivers (FCs) of community-dwelling older adults with dementia. This study used an explanatory mixed methods design in which survey data was augmented with semi-structured interviews. A purposive sample of 20 older adult dyads participated. The intervention consisted of 6-weeks of telephone coaching sessions. FCs conducted daily delirium assessments. We employed correlations and GLM to investigate the relationships between variables and the outcomes. Results: The model showed a statistically significant positive correlation between the Human Connection Scale and the SF-36 pretest domain of general health (r= .47, p= .04). There were statistically significant positive correlations between the Human Connection Scale and the SF-36 posttest domains of physical functioning (r= .54, p= .014) and general health (r= .76, p<.001). There were a small positive changes in mean scores on each domain between the pre- and post-test scores on the SF-36. The most impressive findings came from FCs identification of delirium using the FAM-CAM. These participants had no history of delirium but 6 of 20 (30%) reported at least one episode of delirium. The qualitative data revealed that FGs found weekly coaching sessions beneficial and supportive. The results suggests that the intervention has a meaningful impact on how we assess delirium in the community and warrants further study.

